# Occurrence, health risk of PAHs and the interrelated microbial communities in ‌the sediment of Jinzhou Bay

**DOI:** 10.3389/fmicb.2025.1657904

**Published:** 2025-09-15

**Authors:** Meihua Lian, Yugang Yang, Yaqi Li, Xiaoman Yu

**Affiliations:** ^1^School of Environmental and Chemical Engineering, Shenyang Ligong University, Shenyang, Liaoning, China; ^2^College of Land and Environment, Shenyang Agricultural University, Shenyang, Liaoning, China

**Keywords:** PAH, sediment, estuary, microbial community, risk assessment

## Abstract

Polycyclic aromatic hydrocarbons (PAHs), a class of persistent organic pollutants prevalent in estuarine sediments, were systematically investigated for their spatial distribution, human health risks, and microbial community interactions across selected contamination gradients. Analytical results demonstrated ∑PAH concentrations in sediments ranging from 0.691 to 25.083 mg/kg dry weight, with benzo[a]pyrene toxic equivalents (TEQBaP) exceeding international sediment quality guidelines (ISQGs) at 30% of sampling sites, primarily near anthropogenic emission hotspots. Lifetime carcinogenic risk assessments revealed maximum values of 2.41 × 10^−^⁵ (children), 1.98 × 10^−^⁵ (adolescents), and 3.04 × 10^−^⁵ (adults), with wastewater discharge zones exhibiting both the highest PAH concentrations and population exposure risks. Taxonomic profiling revealed sediment bacterial communities dominated by Proteobacteria, Bacteroidetes and Chloroflexi at the phylum level. The sulfate-reducing genus *Desulfobulbus* was ubiquitously detected except at upstream reference sites. Multivariate redundancy analysis (RDA) revealed that total nitrogen (TN), total phosphorus (TP), total organic carbon (TOC), sulfur (S), and PAHs concentrations constituted the key variables governing microbial community structure (*p* < 0.05).

## 1 Introduction

Estuaries serve as the junction where rivers meet, making them particularly susceptible to the impacts of human activities and the pollutants that are released into the environment (Zeng et al., 2023). Changes in the physical and chemical properties of the water–sediment interface can lead to the re-release of pollutants into the external environment. This transition causes sediments to shift from functioning as a “sink” to becoming a “source” of contaminants, which can result in significant pollution in the estuary area and pose potential ecological risks (Grmasha et al., 2023).

Polycyclic Aromatic Hydrocarbons (PAHs) represent a significant category of persistent organic pollutants, primarily released through the combustion of fossil fuels, industrial discharges, and various anthropogenic activities (Xie et al., 2021). Sixteen PAHs have been identified as priority contaminants by the Environmental Protection Agency (EPA) of the United States (Bouzekry et al., 2024). Low molecular weight PAHs, which consist of 2–3 benzene rings, are classified as non-carcinogens. The high molecular weight PAHs, specifically those with 4 to 6 rings, are classified as carcinogens. Upon release into the environment, PAHs have the potential to migrate over considerable distances, dispersing into soils, sediments, water, and the atmosphere on a global scale (Dreij et al., 2019; Lv et al., 2020). The accumulation of PAHs in sediments over extended periods functions as a primary reservoir. Concentrations of PAHs in the Pearl River estuary and surrounding sea regions varied between 189 and 637 ng/g. The primary sources of pollution were identified as petroleum burning within the estuary and coal combustion in the areas beyond the estuary (Luo et al., 2006). The concentration of PAHs in the surface sediments of the estuarine region of the Pearl River Delta ranged from 69.1 to 1,297 ng/g, indicating a medium level of ecological risk (Li et al., 2021). Research has demonstrated that there is considerable spatial variability of PAHs in sediments between the northern and southern regions, attributed to differences in climate, energy sources, and industrial activities in China. PAHs are oleophilic and hydrophobic, allowing them to accumulate in the human body through inhalation, ingestion, and other routes. Their reputation as a major issue stems from the carcinogenic, mutagenic, and teratogenic impacts on aquatic organisms and human health (Han et al., 2022; Venkatraman et al., 2024). Consequently, recognizing their spatial distributions and assessing the ecological risks are essential for formulating management strategies that are customized to the areas.

The diversity of habitats and environments in estuarine regions, along with the numerous contaminants introduced by river inputs and sewage discharge, support microbial life and elicit varied responses to environmental stressors (Ohore et al., 2022; Qiang et al., 2021). Extended exposure to contamination can lead to the adaptation of certain microorganisms, resulting in alterations to the community structure. The microbial community plays a vital role in nutrient and organic matter cycling and constitutes a significant portion of biomass in sediments (Johnston and Leff, 2015). Previous research has shown that PAH pollution in sediments originating from industrial activities influences the composition and density of microbial communities, and can even disrupt their metabolic processes within the environment (Liu et al., 2018). Recent studies indicate a decrease in bacterial alpha-diversity in response to increased PAH concentrations, attributed to the proliferation of hydrocarbon-clastic organisms (Kimes et al., 2014; Jeanbille et al., 2016). Deltaproteobacteria and Gammaproteobacteria, known for their role in hydrocarbon degradation, are identified as the predominant and frequently occurring microbes in sediments located near coastal regions (Jeanbille et al., 2016). Nevertheless, the reaction of microorganisms to the persistent pollution of PAHs in sediment has not been comprehensively revealed, potentially due to the low concentration of PAHs and the intricate determination process involved. Recent findings indicate that chronic pollution extends exposure duration and enhances community stability, thereby promoting greater diversity within the bacterial community (Nogales et al., 2007; Zhang et al., 2008). Furthermore, it is suggested that environmental factors have a greater influence on microbial communities, indicating that the role of contaminants has been previously concealed and overlooked.

Jinzhou Bay represents a typical example of a heavily polluted coastal area in China, primarily attributed to the activities associated with nonferrous metal smelting industries. Significant quantities of metal(loid) and PAHs were discharged from the Huludao Zn Plant, recognized as one of the largest zinc facilities in Asia, into the estuarine and coastal regions (Li et al., 2012; Zeng et al., 2023). Investigation and analysis revealed that the concentration of PAHs was elevated in the sub-region. Therefore, it is essential to evaluate the occurrence and health risk of PAHs, as well as to identify the microbial community and its diversity in sediments, to comprehend the impacts of regional pollutant disturbances on estuarine ecosystems.

## 2 Materials and methods

### 2.1 Sedimental samples collection, extraction, and analysis

**Figure 1:**
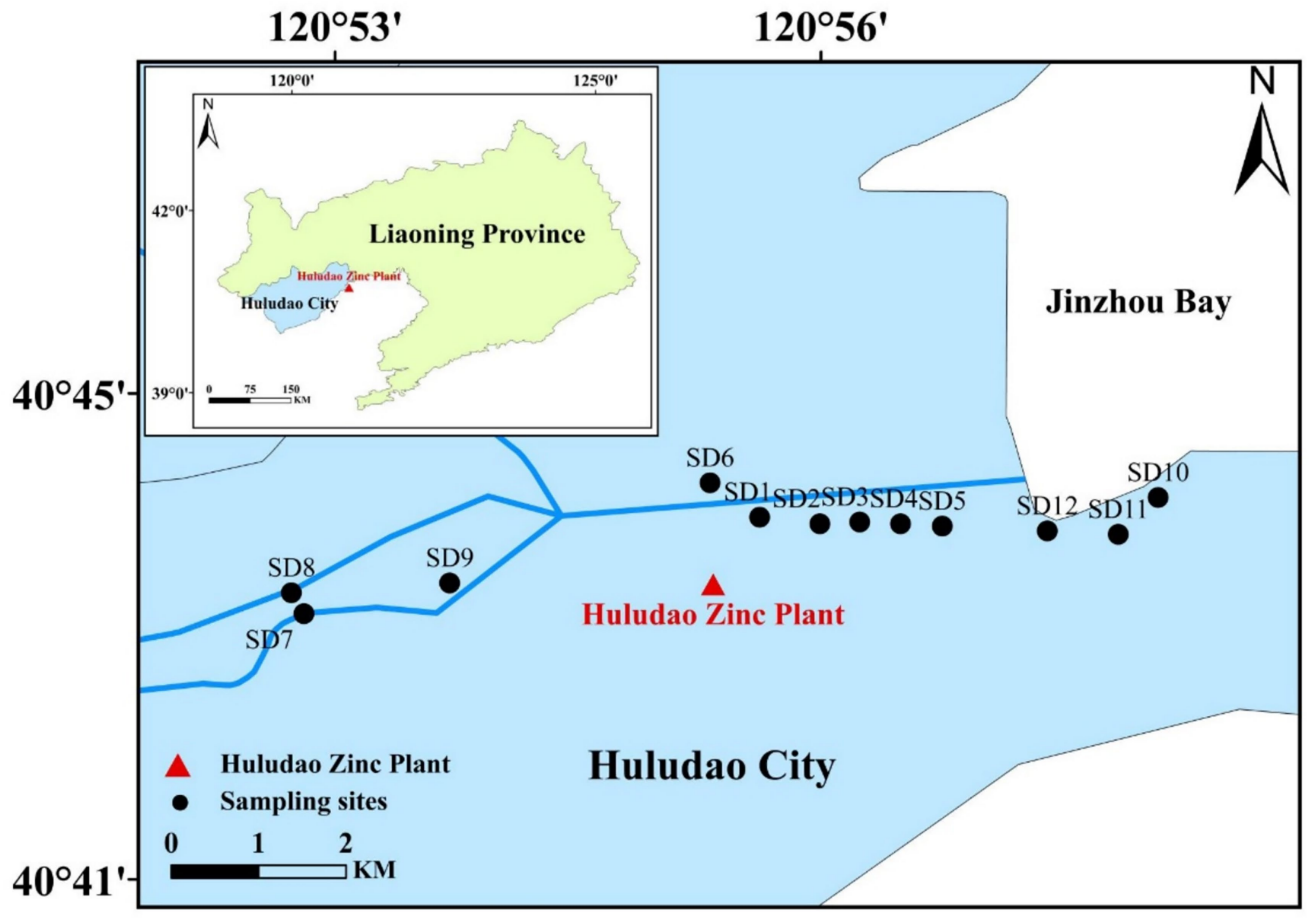
Sampling sites in the typical polluted estuary area.

Figure 1 illustrates sample sites within a typical polluted estuary area, specifically the Huludao Estuary. This study collected a total of 12 points, which primarily encompass the upstream region of Wuli River (SD7, SD8, SD9), the pollutant discharge zone (SD1, SD6), and the downstream area of the sewage outlet (Jinzhou Bay area SD2, SD3, SD4, SD5, SD10, SD11, SD12). Surface sediments were collected using a stainless-steel shovel. Surface samples measuring 0–5 cm were collected and placed into polyethylene sample bags. These bags were then sealed and stored at a temperature of −20 °C. Soil pH was determined by a Sartorius PB-10 pH meter (Sartorius, Germany), while the total concentrations of nitrogen (TN), sulfur (S), and total organic carbon (TOC) were analyzed using a MAT253 isotope ratio mass spectrometer instrument (Thermo Fisher Scientific, Bremen, Germany). Total phosphorus (TP) was determined by the potassium persulfate oxidation method and measured by spectrophotometry (UV-2500, Shimadzu).

### 2.2 Determination of PAHs

Upon returning to the laboratory, the samples underwent freeze-drying, were ground using an agate mortar and sieved to 2 mm. Briefly, 2.0 g of the sieved soil sample were precisely placed in a conical flask and PAHs were extracted with dichloromethane using Soxhlet extractor. Then the extract was purified by passing it through a magnesium silicate purification column, evaporated to dryness by nitrogen gas, redissolved in acetonitrile and transferred to a chromatographic injection bottle for analysis. The concentrations of PAHs in acetonitrile were analyzed by high-performance liquid chromatography (HPLC series 1260s, Agilent) fitted with C18 column. The injection volume was 10 μL and the mobile phase was ultrapure water and acetonitrile (40%: 60%), with column temperature at 30 °C. The flow rate was 0.8 mL/min (Xue et al., 2024). The fluorescence parameters of 16 PAHs, including excitation/emission wavelengths and detection limits were listed in Supplementary Table S1.

An internal calibration procedure with a mixture of PAH standards was used to quantify PAHs concentrations. Calibration standard was analyzed to determine the accuracy of the calibration curves and accuracy of the analytical method. The standard substances used in the experiment were purchased from the AccuStandard company. Recovery of PAHs was assessed by spiking clean sediments at known concentrations and was between 85.6–96.3%.

### 2.3 Toxic evaluation of PAHs

The toxicities of PAHs were assessed by determining the toxicity value for each PAH (Equation 1), utilizing the toxic equivalent concentration of Benzo[a]pyrene (TEQ) in accordance with the toxic equivalent factor (TEFs) (Nisbet and Lagoy, 1992). The concentration of each PAH is converted to the total toxic equivalent concentration (TTEC), which is derived from the *TEQ*_*B*ap_ of each PAHs.


(1) $$\mathrm{TE}Q_{B\mathrm{ap}}=\sum C_{i}\times \mathrm{TE}F_{i}$$

Where, *TEQ*_*B*ap_ is a toxic equivalent concentration, C_i_ is a concentration of PAH_i_, and *TEF*_i_ is a toxicity equivalence factor for each PAH.

### 2.4 Risk assessment models of PAHs

Three essential exposed pathways were selected to compute the chronic daily intakes (CDIs, Equations 2–4). The incremental lifetime cancer risk (ILCR) model serves to assess the health risk associated with PAHs in sediment, as outlined in the Exposure Factor Handbook published by United States Environmental Protection Agency (USEPA) (1999, 2001, 2011) (Equations 5, 6). The model assessed health risks associated with exposure to PAHs in children, adolescents, and adults via ingestion, respiratory inhalation, and skin contact. The potential health risks associated with specific PAHs were assessed through a risk quotient (Cao et al., 2020; Wang et al., 2020), evaluating the likelihood of adverse effects from PAHs in sediments. This assessment involved calculating the TEQ PAHs for molecular weight PAHs, specifically the carcinogenic PAHs: BaA, Chr, BbF, BkF, BaP, InP, and DbA. The TEFs were expressed relative to BaP with values of 0.1, 0.001, 0.1, 0.01, 1, 0.1, and 1, respectively (Adeniji et al., 2019).


(2) $$\mathrm{CD}I_{\text{ingestion}}=\frac{C_{s}\times IR_{s}\times \mathrm{CF}\times \mathrm{EF}\times \mathrm{ED}}{\mathrm{BW}\times \mathrm{AT}}$$

(3) $$\mathrm{CD}I_{\text{inhalation}}=\frac{C_{s}\times \mathrm{HR}\times \mathrm{EF}\times \mathrm{ED}}{{\mathrm{PEF}}_{s}\times \mathrm{BW}\times \mathrm{AT}}$$

(4) $$\mathrm{CD}I_{\text{dermal}}=\frac{C_{s}\times \mathrm{CF}\times \mathrm{AF}\times \mathrm{DAF}\times \mathrm{SA}\times \mathrm{EF}\times \mathrm{ED}}{\mathrm{BW}\times \mathrm{AT}}$$

(5) ILCR=CDI×CSF

(6) $$R=\mathrm{ILC}R_{\text{ingestion}}+\mathrm{ILC}R_{\text{inhalation}}+\mathrm{ILC}R_{\text{dermal}}$$

Where CDI refers to the chronic daily intake associated with ingestion, inhalation, and dermal contact (mg/kg/d). Cs represents the total concentration of converted PAHs in sediment, expressed in terms of toxic equivalents of BaP (mg/kg). CSF is the carcinogenic slope factor (mg/kg/day) and CSF_ingestion_, CSF_dermal_, and CSF_inhalation_ of BaP were considered to be 7.3, 25, and 3.85 (mg/kg/day)^−1^, respectively (Peng et al., 2011). The total cancer risk for the residents (R) is calculated by aggregating the risks associated with various exposure routes of PAHs. The New York State Department of Health categorizes qualitative descriptions of lifetime cancer risks as follows: very low when the estimated value is ≤10^−6^, low from 10^−6^ < R < 10^−4^, moderate from 10^−4^ ≤ R < 10^−3^, high from 10^−3^ ≤ R < 10^−1^ and very high when the value is ≥10^−1^ (NYS DOH, 2012). This study assessed the cancer risk among residents categorized into three groups: children aged 0–10 years, adolescents aged 11–18 years, and adults aged 19–70 years. Other value variables were listed in Supplementary Table S2.

### 2.5 DNA extraction and 16S rRNA gene sequencing

DNA extraction from sediments was performed using the PowerSoil DNA Isolation Kit (MoBio), and the DNA concentration was quantified using a NanoDrop 2000 spectrophotometer (Thermo Fisher Scientific, Waltham, MA, United States). The amplification of 16S rRNA genes was conducted using bacterial-specific primer pairs 338F and 806R, utilizing a thermal cycler PCR system (GeneAmp 9,700, ABI, United States). Amplicons underwent paired-end sequencing at Majorbio located in Shanghai, China. The alpha-diversity indices, including Chao1, Shannon, sobs, and ace were calculated along with the relative abundances of bacterial taxa. Additionally, the significance of environmental factors and PAHs concentrations, in shaping the bacterial community structure was assessed using redundancy analysis (RDA). Origin2021 served as the tool for data processing and the production of charts.

## 3 Results

### 3.1 Concentration of PAHs and environmental parameters in the sediments

**Figure 2:**
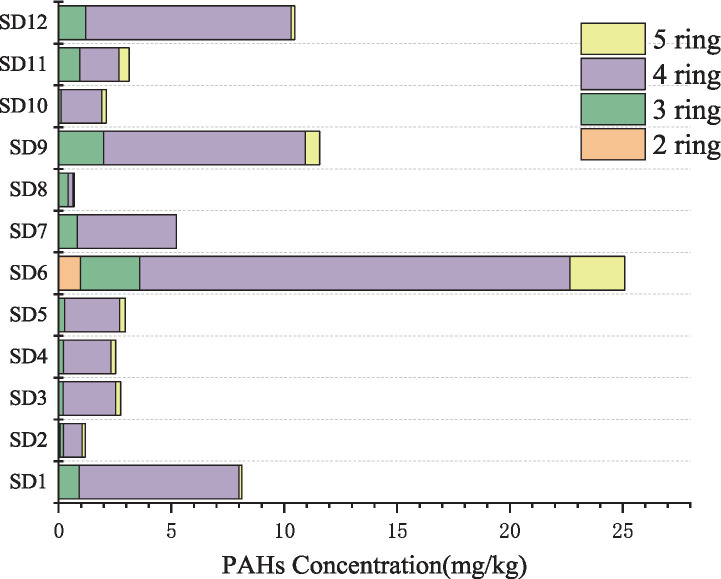
The total PAH concentration in sediments.

The major geochemical properties of the sediments from different sampling sites were investigated. The average concentration of TOC, TN, TP, S, and pH levels in sediment were 27.00 g/kg, 1.47 g/kg, 11.40 mg/kg, 4.50 g/kg, 7.58, respectively. In addition, the total PAH concentration in the sediment samples varied between 0.691 mg/kg and 25.083 mg/kg (Figure 2). The highest concentration was observed in SD6, followed by SD9 at 11.56 mg/kg and SD12 at 10.46 mg/kg. The lowest concentration was recorded in SD8. PAHs are classified into five categories based on the number of benzene rings: two-ring (Nap), three-ring (Ace, Acy, Flu, Phe, and Ant), four-ring (Flt, Pyr, BaA, and Chr), five-ring (BbF, BkF, BaP, and DbA), and six-ring (InP and BghiP) PAHs. Figure 2 illustrates the composition of the categories of PAHs in the samples. The proportions of various rings of PAHs exhibited minor variations. Nap, a two-ring PAH, was detected exclusively at SD2 and SD6, with concentrations measuring 0.067 mg/kg and 0.973 mg/kg, respectively, and was not detected at other locations. The three-ring PAHs comprised 5.45 to 60.8% of the total, with the highest proportion observed in SD8. The concentration and proportion of four-ring PAHs exceeded those of other PAHs, with the proportion at each sampling point ranging from 31.2 to 87.1%. The five-ring PAHs comprised 0 ~ 14.9% of the total content, while the concentration of six-ring PAHs (InP and BghiP) were below the detection limit and not detected in samples.

Furthermore, it is necessary to identify the sources of PAHs for pollution control and remediation. PAH isomer pair ratios such as Ant/(Ant + Phe), Flt/(Flt + Pyr), BaA/(BaA + Chr), and InP/(InP + BghiP) have been widely used to distinguish environmental sources of PAHs. Sources were determined as follows: Ant/(Ant+Phe) ratio <0.1—petroleum/combustion transition point; Flt/(Flt + Pyr) > 0.5—combustion of coal and biomass, and 0.4 ~ 0.5—fossil combustion; BaA/(BaA + Chr) < 0.2—petroleum, 0.2 ~ 0.35—either petroleum or combustion, >0.35—combustion (Lan et al., 2016). Supplementary Table S3 shows the diagnostic ratios (based on the concentrations of PAHs) of Ant/(Ant + Phe), Flt/(Flt + Pyr), BaA/(BaA + Chr) and Phe/Ant. The ratios of Ant/(Phe + Ant) ranged from0.53 to 0.87, and all samples were greater than 0.10, indicating that coal combustion was the main source of the PAHs. Flt/(Flt + Pyr) mainly between 0.4–0.5, while the BaA/(BaA + Chr) and Phe/Ant also suggested the coal and other fossil energy source. Results indicated that the combustion of coal and other fossil fuel from the surrounding zone was the main source of PAH in the study area.

The toxicological assessment was conducted based on the content of 16 PAHs in soil, as detailed in Supplementary Table S4. The findings indicated that the *TEQ*_*B*ap_ values at each sampling location varied between 0.057 and 1.86 mg/kg, with approximately 30% of the sites surpassing the established standard, particularly noted at SD6.

### 3.2 Health risk assessment of PAHs in sediment

**Figure 3:**
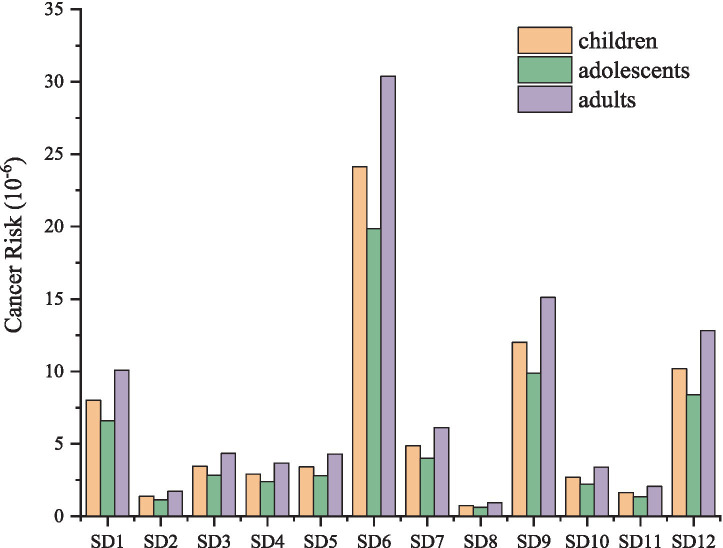
Carcinogenic risk of PAHs in sediments.

Humans are at risk from direct contact with coastal sediments, especially at low tide (Bouzekry et al., 2024). The recreational activities, coastal settlements and fishing are more common in the study environment (Grmasha et al., 2023). The daily exposure dose for three exposure routes was determined by quantifying the concentration of PAHs in sediment across various sampling locations (Table 1). In comparison to respiratory ingestion, both dermal contact and ingestion exhibited similar magnitudes, approximately 10^−7^. The highest daily exposure dose was observed through the ingestion route, with variation ranges for children being 4.51 × 10^−8^ mg/kg/d ~ 1.47 × 10^−6^ mg/kg/d, 1.37 × 10^−8^ mg/kg/d ~ 4.48 × 10^−7^ mg/kg/d, and 4.60 × 10^−8^ mg/kg/d ~ 1.50 × 10^−6^ mg/kg/d, respectively. The exposure dose at SD6 was the highest among the sampling points, with the ingestion exposure doses for three distinct populations recorded as 1.47 × 10^−6^ mg/kg/d, 4.48 × 10^−7^ mg/kg/d, 1.50 × 10^−6^ mg/kg/d. Respiratory intake was significantly lower than ingestion and skin contact, with the lowest daily exposure observed at all sites. Carcinogenic risk assessment indicates that adults face the greatest risk of carcinogenic health effects, followed by children and adolescents (Figure 3). SD6 exhibits the highest cancer risk among all sampling sites, with values of 2.41 × 10^−5^, 1.98 × 10^−5^, 3.04 × 10^−5^ of the risk for children, adolescents and adults, respectively. The carcinogenic risk of other sites is ranked as follows: SD9 > SD12 > SD1 > SD7 > SD3 > SD5 > SD4 > SD10 > SD11 > SD2 > SD8. The health risk for all sites exceeds 1.0 × 10^−6^, with the exception of SD8, indicating the presence of potential health risks in this area. It is essential to ensure that adequate occupational protection measures are implemented for the relevant personnel, particularly for those working in proximity to the zinc plant.

**Table 1:** The daily exposure dose for three exposure routes (mg/kg/d).

Samples	Ingestion*10^−7^			Inhalation*10^−11^			Dermal*10^−7^		
	Children	Adolescents	Adults	Children	Adolescents	Adults	Children	Adolescents	Adults
SD1	4.886	1.487	4.979	1.795	1.752	5.855	1.777	2.202	2.579
SD2	0.837	0.255	0.853	0.308	0.300	1.003	0.304	0.377	0.442
SD3	2.099	0.639	2.139	0.771	0.753	2.516	0.763	0.946	1.108
SD4	1.778	0.541	1.812	0.653	0.638	2.131	0.647	0.802	0.938
SD5	2.080	0.633	2.119	0.764	0.746	2.492	0.756	0.938	1.098
SD6	14.716	4.478	14.995	5.407	5.277	17.634	5.351	6.634	7.767
SD7	2.963	0.902	3.019	1.089	1.062	3.550	1.077	1.336	1.564
SD8	0.451	0.137	0.460	0.166	0.162	0.541	0.164	0.203	0.238
SD9	7.320	2.227	7.458	2.689	2.625	8.771	2.662	3.299	3.863
SD10	1.641	0.499	1.672	0.603	0.589	1.967	0.597	0.740	0.866
SD11	0.996	0.303	1.015	0.366	0.357	1.193	0.362	0.449	0.526
SD12	6.211	1.890	6.329	2.282	2.227	7.443	2.259	2.800	3.278

### 3.3 Bacterial communities and functions in studied areas

**Figure 4:**
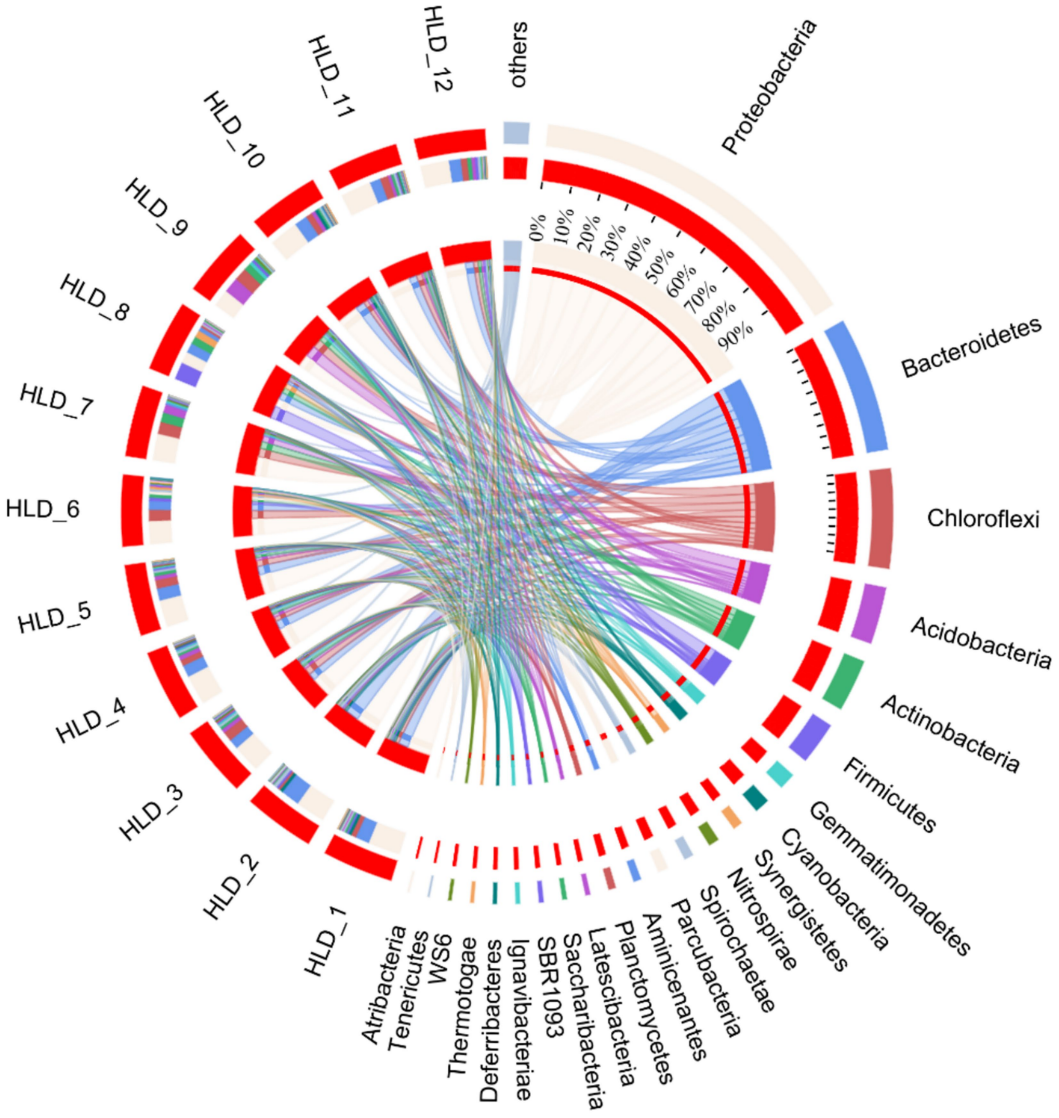
Community composition at the Phylum level in sediment samples. HLD1-HLD12 represents the sites of SD1–SD12.

**Figure 5:**
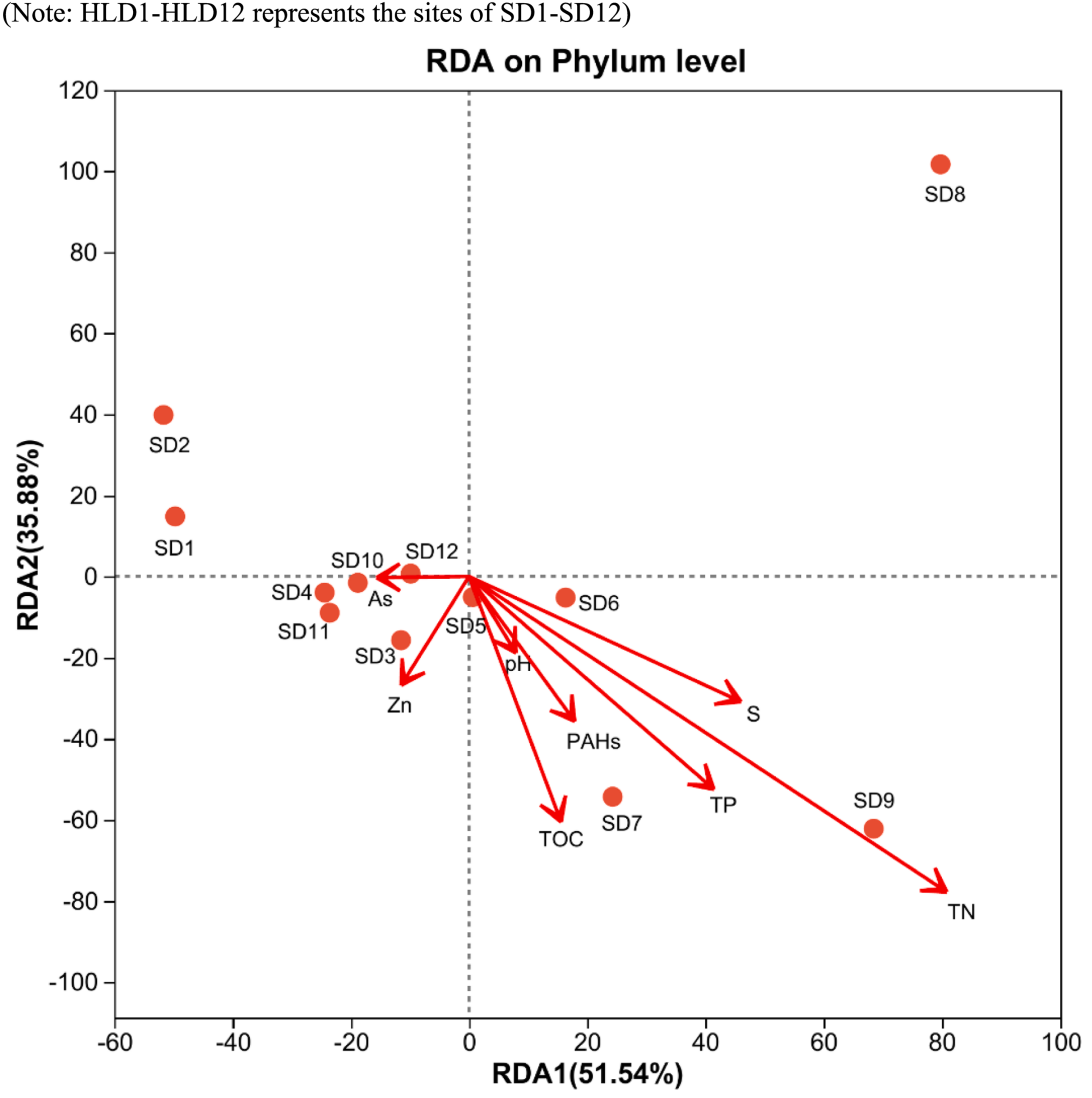
Redundancy analysis of the environmental variables and the composition of microbial communities.

The analysis focused on the diversity and composition of bacterial communities in sediments from estuaries in offshore areas, revealing variations in Alpha diversity among different sites (Supplementary Table S5). The Chao1, Shannon, sobs, and ace metrics of the bacterial communities in SD11 and SD12 were the highest among the sampling areas, whereas SD9 exhibited the lowest values. This indicates that SD11 and SD12 had the greatest bacterial abundance and diversity. In the interim, the composition of microbial communities was investigated, revealing that the bacterial communities predominantly consisted of proteobacteria, bacteroidetes, chloroflexi, acidobacteria, actinobacteria, and firmicutes at the Phylum level in our sediment samples (Figure 4). The highest relative abundance was associated with Proteobacteria, with values ranging from 17.6 to 49.2%. The primary community composition remains consistent with findings from other studies, indicating that Proteobacteria are more prevalent in severely polluted regions compared to areas with lower levels of PAHs (Lin et al., 2023; Ming et al., 2021). At general level, the highest abundances were primarily associated with *Anaerolineaceae, sediment JTB255, and Sulfurimon*as in SD 6 and SD1, which exhibited more severe PAHs pollution. Furthermore, *Desulfobulbus* was identified at all locations except the upstream sites SD7, SD8, and SD9. The sediments of SD1, SD2, and SD6 exhibited a significant concentration of sulfur and the bacterial group of *Sulfurimonas* at the generic level in SD1 and SD6 showed a significantly higher presence compared to other sites, with relative abundances of 9.32 and 7.75%, respectively. RDA was conducted to determine the relationships between environmental variables and the composition of microbial communities in sediment across various sites (Figure 5). The initial axis of the RDA accounted for 51.5% of the variation observed in the surface sediment microbial communities. It exhibited the factors of PAHs, TOC, S, TN, and TP constituted the key variables governing microbial community structure (*p* < 0.05).

## 4 Discussion

River and offshore estuarine sediments are major “sinks” for terrestrial pollutants. The levels of PAHs and the geochemical conditions at the sampling sites, ranging from the upstream of a contaminated river to the estuaries in offshore areas, were assessed and analyzed for comparison. The highest concentration of PAHs was detected at sewage outflows and near the Zn smelting plant, while the levels of PAHs in upstream locations were lower compared to those found in downstream estuaries at offshore sites. Previous study indicates that rivers serve as significant conduits, transporting land-derived sediments that contain contaminants, which can migrate and accumulate in estuarine sediments and offshore marine environments, leading to pollution of organic substances (Wu et al., 2022; Zeng et al., 2023). The levels of PAHs in estuarine sediments ranged from 1 to 251 ng/g, with a mean value of 50.38 ng/g observed in the marine sediments of the Mediterranean (Bouzekry et al., 2024). In contrast, the sediment from the Al-Hussainya River in Karbala Province, Iraq, exhibited concentrations from 0.36 to 119.06 μg/g dw (Hassan et al., 2019). Several sediment cores indicated the presence of PAH contamination “pockets,” displaying levels 200 times greater than those reported in the previous study, and comparable to the findings of the subsequent research. In the areas identified as “hot spots” for PAH-contaminated sediments, the predominant composition of PAHs consisted of three- and four-ring structures. Concentration of two-ring is relatively low primarily because the higher vapor pressure and greater water solubility of LMW PAHs, leading to more rapid volatilization or faster degradation rate (Lan et al., 2016; Murphy et al., 2016; Simo et al., 1997).

Different PAH sources exhibit distinct compositional patterns, and the isomer pair ratios analyzed in this study suggest that coal and fossil fuel combustion are likely the dominant contributors. Geospatial analysis revealed significantly higher PAH concentrations at site SD6 compared to other sampling locations. Given the absence of other high-energy-consuming industries or substantial domestic coal usage in the study area, emissions from the nearby Zn smelting plant are hypothesized to be the primary source of PAHs. This aligns with prior research identifying smelting operations as major emitters of industrial PAHs, often leading to significant PAH accumulation in adjacent soils (Boente et al., 2020). Furthermore, the predominance of 4-ring PAHs in the sediment profile supports their origin from high-temperature combustion processes (Mai et al., 2003), consistent with the industrial activities in the region. It has been reported that the sediments near the industrial area showed greater levels of PAHs than other sites, which may be attributed to variations in coal and fossil fuel combustion in adjacent areas (Zhang et al., 2023).

People living in coastal areas not only consume seafood such as clams directly, but also inevitably come into direct contact with pollutants in the sediment. Activities like fishing and recreational activities can all lead to the entry of these pollutants into the human body through various exposure routes, thereby posing health risks (Bouzekry et al., 2024). The cancer risk associated with PAHs for both children and adults was greater than that for adolescents. Children exhibit heightened sensitivity to environmental pollutants, attributed to their smaller body weight and skin surface area, which increases the likelihood of ingestion through frequent hand-to-mouth activities. Extended exposure to outdoor work or occupations may increase the potential cancer risk for adults (Halfadji et al., 2021). Furthermore, the contaminants present in the sediment are susceptible to influence and release from the surrounding environment, indicating that the associated health risks warrant careful consideration.

Hydrocarbon pollutants have been established as a key driver of microbial community composition and diversity shifts in marine sediments (Lin et al., 2023). Our findings align with Cheng et al. (2024), who reported Proteobacteria (20.6–80.0%) and Bacteroidetes (2.2–65.3%) as the dominant phyla in surface sediments. This phylogenetic distribution suggests that PAH contamination may selectively enrich hydrocarbon-degrading bacterial populations, thereby restructuring microbial communities. Notably, certain indigenous microbial taxa appear particularly adapted to PAH-stressed environments, as evidenced by increased functional gene transcripts and the proliferation of PAH-degrading specialists (Liu et al., 2021). The presence of Bacteroidetes, a phylum previously associated with hydrocarbon contamination in our study sites further supports this adaptive selection hypothesis (Haller et al., 2011). At the genus level, keystone species in our study were predominantly affiliated with Chloroflexi and *δ*-Proteobacteria. *Anaerolineaceae*, a family of Chloroflexi, widely distributed in anaerobic environments (e.g., sediments, wetlands, and wastewater systems) and demonstrated significant PAH degradation potential (Cheng et al., 2024). Within δ-Proteobacteria, the genus *Desulfobulbus* exhibited hydrocarbon degradation capacity under anaerobic conditions. Both *Anaerolineaceae* and *Desulfuromonadales* were closely associated with coupled carbon cycling and iron reduction processes across sediment layers, playing pivotal roles in organic matter mineralization (Coates et al., 2001). Previous studies indicated that *Desulfobulbus* and certain members of these groups are classified as sulfate-reducing bacteria (SRB), primarily resulting from anthropogenic activities (Cleary et al., 2012). Additionally, the class encompassing the majority of sulfate-reducing genera plays a vital role in the anaerobic breakdown of organic matter and is involved in the degradation of PAHs in sediments (Acosta-Gonzalez et al., 2013). Yan et al. (2018) denoted these species whose niche space might be relatively complementary, could respond to anthropogenic disturbances in the co-occurrence networks.

The co-occurrence of contaminants (e.g., PAHs) and key elements (e.g., C, N, P, and S) in sediments exerts complex influences on microbial community composition and function (Yang et al., 2020). RDA identified TOC, sulfur, TN, TP, and PAHs as key determinants shaping bacterial community structure. Notably, Xu et al. (2014) demonstrated that the coexistence of sulfur, nitrate and PAHs can enhance the abundance of functional genes in nitrate-reducing, sulfide-oxidizing bacteria and PAH-degrading microorganisms. Although PAHs are classified as persistent organic pollutants, they can also serve as carbon substrates for microbial metabolism (Cheng et al., 2024), potentially explaining the observed positive correlations between PAH concentrations and the relative abundance of certain microbial genera. Previous studies have well established the critical roles of carbon and phosphorus in governing microbial community structure (Zhang et al., 2023). Liu et al. (2021) further reported that elevated PAH levels, coupled with nitrogen and phosphorus enrichment in heavily polluted black-odor river sediments, may stimulate the growth of specific bacterial taxa and enrich functional genes associated with PAH degradation (nah, nidA) and sulfur reduction (dsrA). Additionally, Lapointe et al. (2005) have previously shown that sewage inputs are a primary source of nitrogen to marine harbor environments, while some sampling sites just located in the downstream area of the sewage discharge outlet. Such anthropogenic inputs can alter nitrogen and carbon cycling, thereby modulating the microbial community’s capacity to degrade pollutants (Atlas and Bartha, 1972; Murphy et al., 2016).

Elevated concentrations of PAHs in sedimentary pose significant ecological risks, potentially altering microbial community structure and impairing key biogeochemical cycling processes of carbon, nitrogen, phosphorus, and sulfur (Xu et al., 2014; Qian et al., 2021). However, current limitations in sequencing-based functional characterization hinder comprehensive understanding of microbial metabolic pathways, environmental adaptation mechanisms, and ecological roles under PAHs stress. Further investigations are warranted to elucidate these functional aspects at molecular level.

## 5 Conclusion

The present research provides a thorough assessment of the levels and ecological risks associated with PAHs, as well as an analysis of the community structure in sediments gathered from estuaries in offshore regions. The total PAH concentration in the sediment samples varied between 0.691 mg/kg and 25.083 mg/kg, and the TEQ_BaP_ values at each sample varied between 0.057 and 1.86 mg/kg, with approximately 30% of the sites surpassing ISQGs of the established standard. The greatest concentration of PAHs and associated health risks were identified at sewage outflow sites, with the highest cancer risk of PAHs were 2.41 × 10^−5^, 1.98 × 10^−5^, 3.04 × 10^−5^ for children, adolescents and adults, respectively. A comprehensive bacterial community was identified through phylogenetic analyzes, with the predominant groups being proteobacteria, bacteroidetes, and chloroflexi. PAH concentrations, in conjunction with sediment nutrient and geochemical parameters, were identified as key determinants shaping microbial community composition.

## Data Availability

The DNA sequencing data presented in this study is available in the National Genomics Data Center (NGDC) Genome Sequence Archive (GSA) under accession number CRA029532, https://ngdc.cncb.ac.cn/gsa/.
