# Supplementary material for: Occurrence, health risk of PAHs and the interrelated microbial communities in ‌the sediment of Jinzhou Bay
Source: Front Microbiol. 2025 Sep 15;16:1657904. doi: 10.3389/fmicb.2025.1657904 (PMC12477186; doi:10.3389/fmicb.2025.1657904)
Supplement: Supplementary file 1 [file Table_1.docx]

Table S1 Fluorescence parameters and the detection limits of PAHs

| Full Names | abbreviations | E_X_（nm） | Em（nm） | LOD（μg/L） | LOQ（μg/L） |
| --- | --- | --- | --- | --- | --- |
| Naphthalene | Nap | 280 | 330 | 0.92 | 2.71 |
| Acenaphthylene | Acy | - | - | - | - |
| Acenaphthene | Ace | 270 | 320 | 0.51 | 1.52 |
| Fluorene | Flu | 280 | 320 | 0.24 | 0.86 |
| Phenanthrene | Phe | 275 | 350 | 0.32 | 1.15 |
| Anthracene | Ant | 260 | 420 | 0.10 | 0.40 |
| Fluoranthene | Flt | 270 | 440 | 0.10 | 0.32 |
| Pyrene | Pyr | 265 | 390 | 0.08 | 0.30 |
| Benz[a]anthracene | BaA | 280 | 420 | 0.04 | 0.12 |
| Chrysene | Chr | 265 | 420 | 0.05 | 0.15 |
| Benzo[b]fluoranthene | BbF | 295 | 440 | 0.05 | 0.15 |
| Benzo[k]fluoranthene | BkF | 290 | 430 | 0.05 | 0.14 |
| Benzo[a]pyrene | BaP | 295 | 430 | 0.04 | 0.17 |
| Dibenz[a,h]anthracene | DbA | 290 | 430 | 0.05 | 0.16 |
| Benzo[g,h,i]perylene | BghiP | 290 | 430 | 0.06 | 0.22 |
| Indeno[1,2,3-cd]pyrene | InP | 280 | 480 | 0.06 | 0.21 |

Table S2 The value of variables collected from literatures

| Definition | Abbreviation | Units | children | adolescents | adult | Reference |
| --- | --- | --- | --- | --- | --- | --- |
| Average body weight | BW | kg | 24.2 | 56.8 | 69.6 | Halfadji, 2021 |
| conversion factor | Csoil | mg/kg | 1.0×10^-6^ | | | USEPA,1999, 2011 |
| Exposure frequency | EF | day/year | 350 | 350 | 350 | USEPA, 1999 |
| Exposure duration | ED | Years | 7 | 10 | 41 | USEPA, 2011 |
| Inhalation rate | HR | m^3^/day | 10 | 16 | 16 | USEPA, 2001, 2011 |
| Soil ingestion rate | IR | mg/day | 200 | 100 | 100 | USEPA, 1999 |
| Dermal surface exposure | SA | cm^2^/day | 2800 | 5700 | 5700 | USEPA, 2001 |
| Dermal adherence factor | AF | mg/cm^2^ | 0.2 | 0.2 | 0.07 | USEPA, 2001 |
| Dermal adsorption fraction | DAF | unitless | 0.13 | | | USEPA, 2001 |
| Average time for lifetime span | AT | days | 365×70 | | | USEPA, 2001 |
| Particulate emission factor | PEF | m^3^/kg | 1.36×10^9^ | | | USEPA, 2001 |

Table S3 Diagnostic ratios of PAHs

| Sample | Ant/(ant+phe) | Fla/Fla+Pyr | BaA/BaA+Chr | Phe/Ant |
| --- | --- | --- | --- | --- |
| SD 1 | 0.79 | 0.48 | 0.98 | 0.27 |
| SD 2 | 0.62 | 0.53 | 0.98 | 0.60 |
| SD 3 | 0.77 | - | 0.99 | 0.28 |
| SD 4 | 0.79 | 0.43 | 0.99 | 0.26 |
| SD 5 | 0.81 | 0.47 | 0.99 | 0.24 |
| SD6 | 0.87 | 0.48 | 0.98 | 0.15 |
| SD 7 | 0.74 | 0.48 | 1.00 | 0.36 |
| SD8 | 0.78 | - | - | 0.27 |
| SD 9 | 0.76 | 0.46 | 0.998 | 0.31 |
| SD10 | 0.79 | 0.43 | 0.99 | 0.25 |
| SD11 | 0.84 | 0.48 | 0 | 0.19 |
| SD12 | 0.82 | 0.49 | 0.98 | 0.22 |

Table S4 TEQBaP values of sampling sites

| PAH | TEF | SD1 | SD 2 | SD 3 | SD 4 | SD 5 | SD 6 | SD 7 | SD 8 | SD 9 | SD 10 | SD 11 | SD 12 |
| --- | --- | --- | --- | --- | --- | --- | --- | --- | --- | --- | --- | --- | --- |
| Nap | 0.001 | - | 6.70E-05 | - | - | - | 9.73 E-04 | - | - | - | - | - | - |
| Acy | 0.001 | - | - | - | - | - | - | - | - | - | - | - | - |
| Ace+ Flu | 0.001 | 1.83 E-04 | 2.91E-05 | 3.24E-05 | 4.07E-05 | 3.88E-05 | 4.43 E-04 | 1.35 E-04 | 1.03 E-04 | 9.99E-05 | - | 1.15 E-04 | 1.03 E-04 |
| Phe | 0.001 | 5.35 E-04 | 1.01 E-04 | 1.29E-04 | 1.28 E-04 | 1.59 E-04 | 1.30 E-03 | 5.49 E-04 | 2.32 E-04 | 5.59 E-04 | 8.27E-05 | 5.42 E-04 | 7.62 E-04 |
| Ant | 0.01 | 2.00 E-03 | 1.68 E-04 | 4.48 E-04 | 5.00 E-04 | 6.72 E-04 | 8.80 E-03 | 1.54 E-03 | 8.52 E-04 | 1.81 E-03 | 3.26 E-04 | 2.86 E-03 | 3.38 E-03 |
| Flt | 0.001 | 4.97 E-04 | 8.97E-05 | - | 1.33 E-04 | 1.79 E-04 | 2.18 E-03 | 3.20 E-04 | 0 | 5.52 E-04 | 1.22 E-04 | 6.69 E-04 | 7.00 E-04 |
| Pyr | 0.001 | 5.27 E-04 | 8.06E-05 | 1.72 E-04 | 1.74 E-04 | 2.03 E-04 | 2.36E-03 | 3.48E-04 | 2.15 E-04 | 6.50 E-04 | 1.62 E-04 | 7.22 E-04 | 7.29 E-04 |
| BaA | 0.1 | 0.529 | 5.80 E-02 | 0.197 | 0.164 | 0.196 | 1.292 | 0.371 | - | 0.731 | 0.148 | - | 0.676 |
| Chr | 0.01 | 7.63 E-03 | 7.80 E-04 | 1.77E-03 | 1.60 E-03 | 9.92 E-04 | 1.60 E-02 | - | - | 4.33 E-03 | 3.48 E-04 | 3.46 E-03 | 9.18 E-03 |
| BbF | 0.1 | - | 6.01 E-03 | 9.93 E-03 | 1.00 E-02 | 1.20 E-02 | 0.129 | - | - | 3.70 E-02 | 1.10 E-02 | 2.90 E-02 | - |
| BkF | 0.1 | 5.28 E-03 | 2.31 E-03 | 3.33E-03 | 3.07 E-03 | 3.61 E-03 | 2.67 E-02 | - | - | 1.17 E-02 | 4.00 E-03 | 7.00 E-03 | 7.00 E-03 |
| BaP | 1 | 7.20 E-02 | 3.60 E-02 | 4.90 E-02 | 4.29 E-02 | 4.50 E-02 | 0.324 | - | 5.60 E-02 | 0.136 | 4.20 E-02 | 8.10 E-02 | 8.70 E-02 |
| DbA | 0.1 | - | 2.11 E-03 | 2.98 E-03 | 2.74 E-03 | 4.01 E-03 | 5.34 E-02 | - | - | - | - | - | - |
| TEQ | -- | 0.617 | 0.106 | 0.265 | 0.225 | 0.263 | 1.858 | 0.374 | 0.057 | 0.924 | 0.207 | 0.126 | 0.784 |

Table S5 Variations in Alpha diversity among different sites

| Sample | ace | chao | coverage | shannon | simpson | sobs |
| --- | --- | --- | --- | --- | --- | --- |
| SD1 | 3767 | 3664 | 0.9709 | 6.0701 | 0.00879 | 2655 |
| SD 2 | 3062 | 3084 | 0.9764 | 5.9304 | 0.00828 | 2188 |
| SD 3 | 3758 | 3738 | 0.9691 | 6.3816 | 0.00460 | 2674 |
| SD 4 | 3850 | 3855 | 0.9681 | 6.3957 | 0.00497 | 2765 |
| SD 5 | 3756 | 3708 | 0.9636 | 6.3689 | 0.00506 | 2644 |
| SD 6 | 3402 | 3391 | 0.9692 | 5.9604 | 0.00939 | 2341 |
| SD 7 | 3008 | 3084 | 0.9793 | 6.7221 | 0.00316 | 2443 |
| SD 8 | 3353 | 2875 | 0.9809 | 5.5582 | 0.01469 | 2011 |
| SD 9 | 19283 | 1921 | 0.9845 | 5.8245 | 0.00797 | 1461 |
| SD 10 | 3624 | 3652 | 0.9677 | 6.3921 | 0.00521 | 2617 |
| SD 11 | 4208 | 4296 | 0.9648 | 6.5385 | 0.00410 | 2977 |
| SD 12 | 4059 | 4068 | 0.9637 | 6.4674 | 0.00436 | 2873 |
